# Direct identification of neoantigen-specific TCRs from tumor specimens by high-throughput single-cell sequencing

**DOI:** 10.1136/jitc-2021-002595

**Published:** 2021-07-28

**Authors:** Yong-Chen Lu, Zhili Zheng, Frank J Lowery, Jared J Gartner, Todd D Prickett, Paul F Robbins, Steven A Rosenberg

**Affiliations:** 1 Surgery Branch, National Institutes of Health, Bethesda, Maryland, USA; 2 Department of Pathology and Winthrop P. Rockefeller Cancer Institute, University of Arkansas for Medical Sciences, Little Rock, Arkansas, USA

**Keywords:** immunotherapy, adoptive, lymphocytes, tumor-infiltrating, melanoma, gastrointestinal neoplasms, cell engineering

## Abstract

**Background:**

Recognition of neoantigens by T cells plays a major role in cancer immunotherapy. Identification of neoantigen-specific T-cell receptors (TCRs) has become a critical research tool for studying T cell-mediated responses after immunotherapy. In addition, neoantigen-specific TCRs can be used to modify the specificity of T cells for T cell-based therapies targeting tumor-specific mutations. Although several techniques have been developed to identify TCR sequences, these techniques still require a significant amount of labor, making them impractical in the clinical setting.

**Methods:**

Thanks to the availability of high-throughput single-cell sequencing, we developed a new process to isolate neoantigen-specific TCR sequences. This process included the isolation of tumor-infiltrating T cells from a tumor specimen and the stimulation of T cells by neoantigen-loaded dendritic cells, followed by single-cell sequencing for TCR and T-cell activation markers, interferon-γ and interleukin-2.

**Results:**

In this study, potential neoantigen-specific TCRs were isolated from three melanoma and three colorectal tumor specimens. These TCRs were then synthesized and transduced into autologous T cells, followed by testing the recognition of neoantigens. A total of 28 neoantigen-specific TCRs were identified by this process. If identical TCR sequences were detected from two or more single cells, this approach was highly reliable (100%, 19 out of 19 TCRs).

**Conclusion:**

This single-cell approach provides an efficient process to isolate antigen-specific TCRs for research and clinical applications.

## Background

Cancer immunotherapy has shown to be effective for patients with selected types of cancer.^1 2^ The post-treatment analyses of adoptive cell therapy (ACT) and immune checkpoint blockade therapies have suggested that effective cancer immunotherapies are strongly associated with the activation of neoantigen-reactive T cells.^3–13^ However, the majority of patients with common epithelial cancers do not respond to current immunotherapies, including ACT.^14^ We and others have hypothesized that we may achieve higher efficacy of ACT using cell infusion products containing a higher percentage of diverse neoantigen-reactive T cells with a younger phenotype.^7 13 15 16^ Therefore, we have developed and initiated a new ACT clinical trial (NCT03412877) using autologous peripheral blood T cells transduced with neoantigen-specific T-cell receptors (TCRs) isolated from resected tumors of a patient with cancer.

One of the major bottlenecks for this new type of treatment is the ability to isolate neoantigen-specific TCRs from tumor specimens. Additionally, the same technique can be utilized to research the mechanisms of antitumor responses mediated by neoantigen-reactive T cells. The conventional technique employing T-cell cloning and Sanger sequencing is labor-intensive, time-consuming and technically challenging.^17^ In short, the challenges come from the diversity and complicated biology of TCR.^18^ Some TCRs are difficult to be amplified by PCR, followed by the Sanger sequencing. In addition, up to one third of mature T cells may express two functional TCRα chains, but only one of the two TCRα chains likely contributes to the anticipated specificity.^19^ Lastly, TCRα and β chains from each individual T cell must be paired correctly. Incorrectly paired TCR may lose specificity or gain unwanted specificities.^20^

Previously, we developed a single-cell approach to identify neoantigen-specific TCRs from long-term tumor-infiltrating lymphocyte (TIL) cultures.^17^ A tumor resected from a patient was cut into 24 fragments. These tumor fragments were cultured for about 4 weeks in culture medium containing interleukin-2 (IL-2). Once sufficient numbers of TILs were obtained, a large-scale screening assay was performed to identify neoantigen-reactive TIL cultures and the corresponding neoantigen pools. We then picked neoantigen-reactive TIL cultures and stimulated them with neoantigen-loaded dendritic cells (DCs). We utilized a previous-generation, low-throughput single-cell approach to obtain whole-transcriptome data from the stimulated TILs. Lastly, paired TCR sequences were obtained from the whole-transcriptome data by a bioinformatic approach. Under this experimental condition, we found that the expression levels of interferon-γ (IFN-γ) and IL-2 were two markers to precisely identify neoantigen-specific TCRs. The major limitation of our previous approach was that it required about four labor-intensive weeks to expand TILs from tumor fragment cultures. More importantly, the polyclonal TILs might differentially expand after the long-term culture, leading to the potential loss of some neoantigen-reactive clonotypes. Additionally, efficient high-throughput single-cell analysis had not been developed at that time, limiting our ability to investigate low-frequency clonotypes. To avoid the need of the long-term TIL culture and to take advantage of the newly available high-throughput single-cell technology, we aimed to develop a new approach to isolate neoantigen-specific TCRs directly from tumor specimens. In this report, neoantigen-specific TCRs were obtained from tumor specimens resected from three patients with melanoma and three patients with colorectal cancer. These TCRs were synthesized and transduced into autologous T cells to test their specificities against neoantigens.

## Methods

### Patients

Patients with metastatic cancers were enrolled in clinical trials of TIL immunotherapy at Surgery Branch, National Cancer Institute (ClinicalTrial.gov ID: NCT02621021 and NCT01174121). These trials were approved by the Institutional Review Board of the National Cancer Institute, and written informed consent was obtained from patients, following NIH guidelines and Declaration of Helsinki.

### Isolation of tumor-infiltrating T cells directly from tumor specimens

Tumor specimens were resected from three patients with metastatic melanoma ((patient ID: 4256 (M1), 4261 (M2) and 4202 (M3)) and three patients with metastatic colorectal cancer ((patient ID: 4342 (CC1), 4298 (CC2) and 4254 (CC3)). The majority of the tumor specimens were used to generate long-term TIL cultures for patient treatments. Small portions of the tumor specimens, approximately 1–2 g, were cut into small pieces (2–3 mm) and transferred to gentleMACS C Tubes (Miltenyi Biotec, Germany) with 10 mL RPMI medium per tube containing 10% human AB serum, 0.13 U/mL Liberase TM (Roche/Sigma-Aldrich, St. Louis, Missouri, USA), 600 U/mL DNase I (Roche/Sigma-Aldrich). The C Tubes were loaded on a gentleMACS Dissociator (Miltenyi Biotec), and the tumor specimens were dissociated by selecting h_tumor_01, h_tumor_02 and h_tumor_03 programs on the gentleMACS Dissociator. The C Tubes were incubated at 37°C for 30 min between each program. At the end of the programs, the single-cell suspensions were passed through a 40 µm cell strainer to remove debris. Lastly, the single-cell suspensions were washed once with phosphate-buffered saline (PBS) containing 5 mM EDTA and then cryopreserved.

The single-cell suspension samples containing tumor-infiltrating T cells were thawed and cultured overnight in the RPMI medium with 10% human AB serum, supplemented with a cytokine/antibody/inhibitor cocktail containing 6000 IU/mL IL-2^21^ (Clinigen, Yardley, Pennsylvania, USA), 10 ng/mL IL-15^22^ (Peprotech, Rocky Hill, New Jersey, USA), 10 ng/mL IL-21^22 23^ (Peprotech), 1 µg/mL anti-PD-1 antibody (pembrolizumab, Merck, Kenilworth, New Jersey, USA), 1 µM AKT inhibitor VIII^24^ (Santa Cruz Biotech, Dallas, Texas, USA), 3 µM TGFβRI inhibitor^25 26^ (Galunisertib/ LY2157299, Santa Cruz Biotech). After treatment with DNase (600 U/mL) for 30 min at 37°C, the cells from the single-cell suspension were harvested and stained with Fc Block (BD Biosciences) and 7-AAD (BD Biosciences), followed by anti-PD-1 antibody (Merck). Our previous studies have shown that PD-1 marker could be used to enrich neoantigen-reactive T-cell populations.^27–29^ The anti-PD-1 antibody was detected by biotin-labeled antihuman IgG4 antibody (BD Biosciences), followed by streptavidin-APC (Miltenyi Biotec). CD4^+^ and CD8^+^ T cells were detected by anti-CD4 and CD8 antibodies (BD Biosciences). After staining and washing, CD4^+^PD1^+^ and CD8^+^PD1^+^ T cells were sorted by BD FACSAria II (BD Biosciences). Sorted T cells (1×10^5^ cells/well) were cultured with RPMI medium containing the same cocktail in a 96-well plate for 1 day for T cells from colorectal cancer specimens or 7 days for T cells from melanoma specimens. T cells were then cultured in RPMI medium without the cocktail for additional 16 hours, except CC2. T cells were harvested and resuspended in RPMI medium without any cytokine prior to the neoantigen stimulation.

### Identification of non-synonymous mutations

The process of identifying non-synonymous mutations from tumor specimens has been published previously.^30^ Briefly, genomic DNA was purified from a patient’ tumor specimen and peripheral blood mononuclear cells (PBMCs) using an AllPrep DNA/RNA kit (Qiagen, Germantown, Maryland, USA). Whole-exome libraries were prepared using a SureSelectXT Target Enrichment kit with the human all Exon V7 RNA bait, according to the manufacturer’s protocol (Agilent, Santa Clara, California, USA). Subsequently, whole-exome libraries were sequenced by a NextSeq 550 sequencer using a High-output v2 300-cycle kit (Illumina, San Diego, California, USA). Once the sequencing data were obtained, alignments to human genome build hg19 were performed using novoalign MPI from novocraft (http://www.novocraft.com/). Next, Varscan2 (http://varscan.sourceforge.net) was used to call somatic mutations using the following criteria: tumor and normal read counts of 10 or greater, variant allele frequency of 10% or greater and tumor variant reads of 4 or more. These mutations were then annotated using Annovar (http://annovar.openbioinformatics.org). The data of non-synonymous mutations identified here were used to generate a tandem minigene (TMG) library or a peptide library for neoantigen identification, as described previously.^5^ Because the limited numbers of tumor-infiltrating T cells were obtained from colorectal cancer specimens, only the top-ranked mutations were studied (online supplemental excel file).

10.1136/jitc-2021-002595.supp1 Supplementary data

### Identification of neoantigen-specific TCR sequences by single-cell sequencing

CD14^+^ monocytes were purified from autologous PBMCs by using the IMag antihuman CD14 Magnetic Particles, according to the manufacturer’s protocol (BD Biosciences). About 1×10^7^ CD14^+^ monocytes were then cultured in 10 mL RPMI medium supplemented with 10% fetal calf serum, 50 ng/µL GM-CSF (R&D Systems, Minneapolis, Minnesota, USA) and 20 ng/µL IL-4 (R&D Systems) in a Petri dish. Additional 5 mL medium containing granulocyte-macrophage colony-stimulating factor (GM-CSF) and IL-4 was added on day 3. Loosely adherent DCs were harvested on day 7.

Melanoma specimens contained high numbers of mutations. Therefore, high numbers of peptide pools (PPs) and TMGs were required to test all mutations. To reduce the numbers of PPs and TMGs for the subsequent single-cell analysis, optional screening assays were performed. The day prior to the T-cell stimulation, 1×10^5^ autologous DCs were pulsed with individual PPs or TMG RNAs, and then cultured for 24 hours in a 96-well plate. These neoantigen-loaded DCs were cocultured with about 1×10^5^ tumor-infiltrating T cells per well for 16 hours. After coculture, the levels of IFN-γ secreted by T cells were determined by ELISA. The additional screening assays took 9 days to thaw, isolate and culture tumor-infiltrating T cells, and then 2 days for the actual screening assay. One to three PPs or TMGs were selected for the subsequent single-cell analysis, and the cost of single-cell sequencing was reduced as a result.

The day prior to the T-cell stimulation, 1×10^5^ autologous DCs were pulsed with individual PPs or TMG RNAs, and then cultured overnight in a poly-d-lysine treated 96-well plate. To stimulate T cells, approximately 1–1.5×10^5^ T cells were cocultured with neoantigen-loaded DCs for 4 hours. After stimulation, the non-adherent T cells were harvested and resuspended in PBS at exactly 5×10^5^ cells/mL, and then loaded to a Chromium Controller (10X Genomics, Pleasanton, California, USA) for single-cell sample preparation.

A single-cell V(D)J reagent kit and a Chromium Controller were used to prepare single-cell samples for sequencing, following the manufacturer’s protocol (10X Genomics). Briefly, 10,000 stimulated TILs per channel were loaded on the Chromium Controller, with the targeted cell recovery of 6000 single cells. Two channels were loaded for each stimulated sample. The pooled single-cell cDNA samples were first universally amplified by a 14-cycle PCR, and TCR sequences were enriched by two additional PCRs using TCR-specific primers, according to the manufacturer’s protocol. The pooled samples were sequenced by an Illumina NextSeq 550 sequencer (Read1: 150 b.p. Read2: 150 b.p).

Separately, the T-cell markers were enriched by a PCR from the same pooled single-cell cDNA samples. The following in-house designed primers were used:

IFN-γ 3′ primer (5′-GTCTCGTGGGCTCGGAGATGTGTATAAGAGACAGNGTTTGAAGTAAAAGGAGACAATTTG-3′); IL-2 3′ primer (5′-GTCTCGTGGGCTCGGAGATGTGTATAAGAGACAGNTTCTACAATGGTTGCTGTCTCA-3′);

CD4 3′ primer (5′-GTCTCGTGGGCTCGGAGATGTGTATAAGAGACAGNCTTCTATCTTAAGATTCTTGATGATCA-3′);

CD8A 3′ primer (5′-GTCTCGTGGGCTCGGAGATGTGTATAAGAGACAGNGCAGGAGCAAGGCGGTCACTGGTAAGG-3′);

CD8B 3′primer (5′-GTCTCGTGGGCTCGGAGATGTGTATAAGAGACAGNCTTCCGGCTTCACGCTTGTGAGATTGA-3′). Additionally, a Universal 5′ Primer (5′-ACACTCTTTCCCTACACGACGCTCTTCCGATCT-3′) (Illumina proprietary) was used. These primers were mixed with the pooled single-cell cDNA samples, and a 20-cycle PCR was performed with the annealing temperature at 65°C. Lastly, a Universal-P5 5′ primer (5′-AATGATACGGCGACCACCGAGATCTACACTCTTTCCCTACACGACGCTCTTCCGATCT-3′) (Illumina proprietary) and a 3′ index primer Nextera V2 N70x (Illumina) were used for an additional 10-cycle PCR with the annealing temperature at 65°C. The last PCR was necessary to add adaptors for Illumina next-generation sequencing (Illumina proprietary). The pooled samples were sequenced by an Illumina MiSeq sequencer (Read1: 26 b.p. Read2: 100 b.p).

### Bioinformatic analysis for single-cell sequencing data

Both the in-house T-cell marker and TCR sequencing data were first processed by Cell Ranger pipelines (v2.1.1; 10X Genomics). Full-length TCR sequences, excluding the constant regions, were obtained by Loupe VDJ Browser (10X Genomics). The CDR3β nucleotide sequences are highly diverse, which can be used as natural barcodes.^18^ To streamline the data analysis, the identical T-cell clonotypes and TCRs were defined by the identical CDR3β nucleotide sequences.^31^ T-cell marker sequencing data were mapped to the reference genome database (hg19), and the levels of T-cell marker gene expression were calculated based on the counts of unique molecular identifiers (UMIs). Single cells with high IFN-γ or IL-2 were further analyzed, and barcodes associated with these single cells were obtained. Because each single cell contained a unique barcode, paired TCRα/β sequences from each individual IFN-γ^+^ or IL-2^+^ single cells were obtained based on their matched barcodes. For CD4 and CD8 markers, two or more CD4, CD8A or CD8B UMIs detected from individual single cell were considered as positive. To calculate the frequencies of identified TCR clonotypes, single-cell data generated from each tumor specimen were combined together and reanalyzed. The types and frequencies of TCR clonotypes were determined by the Cell Ranger pipelines. The majority of TCR clonotypes that were only detected once were likely errors, and these singular clonotypes were removed from the frequency calculation.

### Validation of neoantigen-specific TCRs

The detailed protocol has been described previously, with some minor modifications described here.^17 32^ Full-length TCRα and TCRβ sequences with modified mouse constant regions, linked by a furinSGSGP2A linker (rakrsgsgatnfsllkqagdveenpgp), were synthesized and cloned into a MSGV retroviral expression vector.^33–35^ A 1.5 µg of MSGV-TCR plasmid and 0.75 µg of Vesicular stomatitis virus glycoprotein (VSV-G) (RD114) plasmid were cotransfected into 1×1 0^6^ 293 GP cells in each 6-well using Lipofectamine 2000 Transfection Reagent (Invitrogen/Thermo Fisher Scientific, Waltham, Massachusetts, USA). After 48 hours, the supernatant was harvested and spun at 3000 rpm for 10 min to remove debris. The retroviral supernatant was loaded on RetroNectin (Takara, Otsu, Japan) coated 6-well plates by centrifugation at 2000g for 2 hours.

Separately, 1×10^6^/mL PBMCs from healthy donors were stimulated with 50 ng/mL anti-CD3 antibody (clone OKT3) and 1200 IU/mL IL-2 in AIM V medium containing 5% human AB serum. After 2 days, stimulated cells were harvested and resuspended in the same medium without the anti-CD3 antibody. Stimulated PBMCs were added to each retrovirus-loaded well at 2×10^6^ cells/well and spun at 1000 g for 10 min. Plates were incubated overnight at 37°C. On the next day, the PBMCs were transferred to new retrovirus-loaded wells and the transduction procedure was repeated. TCR-transduced T cells were continuously cultured in AIM V medium with 1200 IU/mL IL-2% and 5% human AB serum for five additional days before performing coculture experiments.

To test the specificity of TCR-transduced T cells, autologous DCs were pulsed with 25-mer peptides for 24 hours. About 1×10^5^ T cells were then cocultured with 1×10^5^ autologous DCs overnight in a 96-well U-bottom plate. The supernatant was harvested, and the secretion of IFN-γ from T cells was determined by an ELISA (Thermo Fisher Scientific).

## Results

### The workflow of identifying neoantigen-specific TCRs directly from a tumor specimen

**Figure 1:**
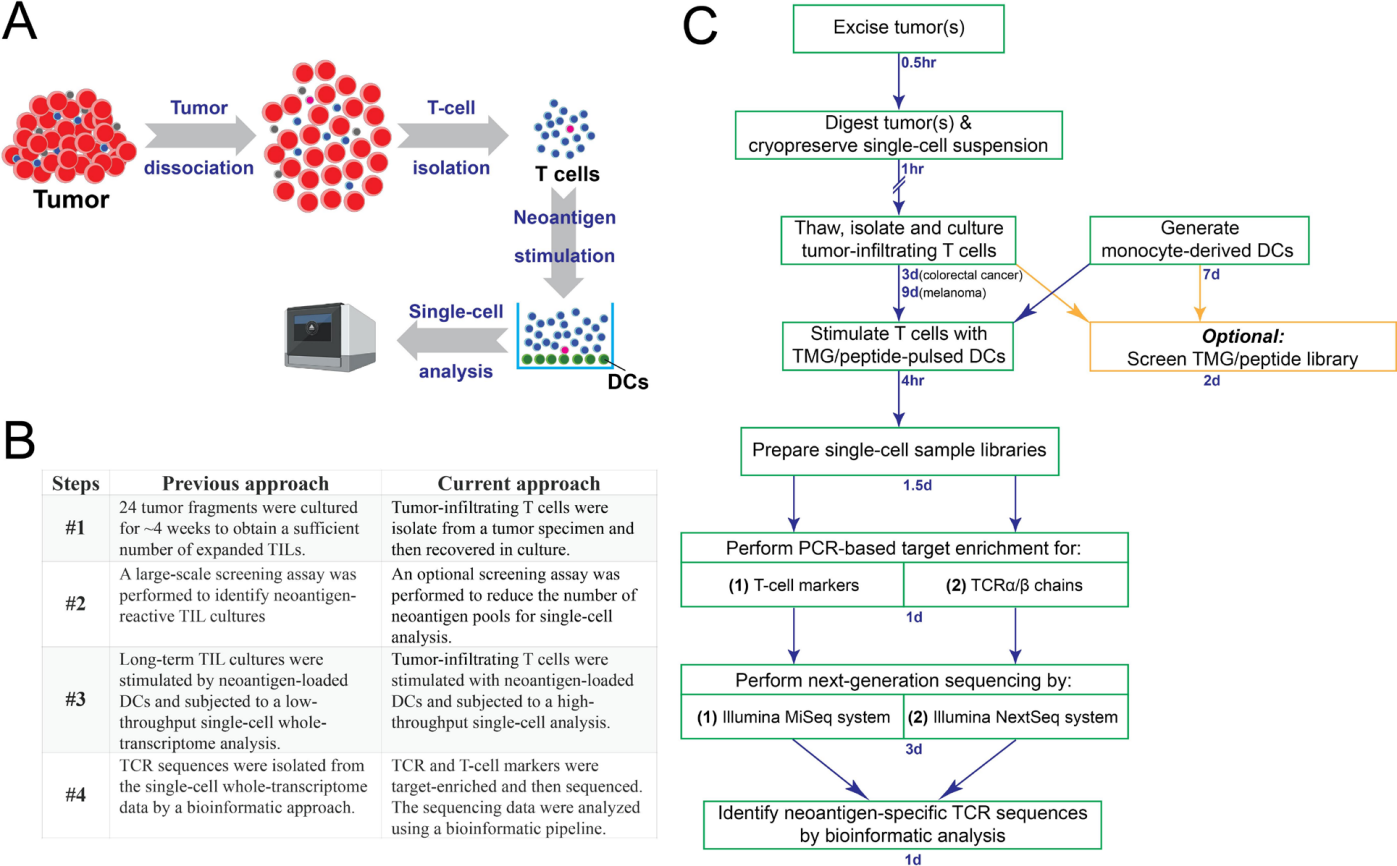
The schema of a new approach to identify neoantigen-specific TCRs directly from a tumor specimen. (A) A brief summary of this newly developed single-cell approach. (B) A comparison between the previous single-cell approach and the current approach. (C) The tumor specimen was digested and cryopreserved. After thawing and recovering, tumor-infiltrating T cells were isolated by fluorescence-activated cell sorting (FACS). In order to reduce the number of single-cell samples for the cost-saving purpose, it is optional to perform screening using a TMG or a peptide library to identify TMGs or peptide pools that could potentially be recognized by these T cells. Next, these polyclonal T cells were stimulated by TMG/peptide-loaded DCs for 4 hours, and then subjected to single-cell sample preparation, followed by PCR-based target enrichment, next-generation sequencing and bioinformatic analysis. DCs, dendritic cells; TCRs, T-cell receptors; TMG, tandem minigene.

In this study, we developed a new approach to identify neoantigen-specific TCRs directly from a tumor specimen. This process included the isolation of tumor-infiltrating T cells from a tumor specimen. T cells were then stimulated by neoantigen-loaded DCs. Single-cell sequencing was performed to identify neoantigen-specific TCR sequences (figure 1A). Although superficially similar to our previous approach,^17^ the new approach differed in several key steps (figure 1B). In our previous approach, the neoantigen-specific TCRs were identified through the following key steps: (1) Approximately 24 tumor fragments were cultured for about 4 weeks in culture medium containing IL-2, in order to obtain a sufficient number of expanded TILs. (2) A large-scale screening assay was performed to identify neoantigen-reactive TIL cultures and also the corresponding neoantigen pools. (3) Based on positive screening results, neoantigen-reactive TIL cultures were then stimulated by neoantigen-loaded DCs and subjected to a low-throughput, single-cell whole-transcriptome analysis. (4) Lastly, TCR sequences were isolated from the single-cell whole-transcriptome data by a bioinformatic approach. In contrast to the previous approach, neoantigen-specific TCRs were isolated through the following key steps: (1) Tumor-infiltrating T cells were sorted from a tumor specimen and then recovered in culture. (2) To reduce the cost associated with single-cell sequencing, an optional screening assay was performed to reduce the number of neoantigen pools for the following single-cell analysis. (3) These T cells were stimulated with neoantigen-loaded DCs and subjected to a high-throughput single-cell analysis. (4) To improve sensitivity, the TCR and T-cell markers were target- enriched by PCR and then sequenced. The sequencing data were analyzed using a bioinformatic pipeline.

The more detailed, step-by-step approach to directly isolate neoantigen-specific TCRs is shown in figure 1C. A tumor specimen was resected from a patient with cancer, and then dissociated into a single-cell suspension, which was cryopreserved until other reagents, such as autologous DCs and a TMG library, were generated. Following the steps described in the Methods section, the single-cell suspension was thawed and recovered overnight, followed by FACS sorting to isolate tumor-infiltrating T cells. In general, a higher number of non-synonymous mutations were identified from melanoma specimens, compared with colorectal cancer specimens. To reduce the number of samples for single-cell sequencing analyses, screening assays were performed for melanoma specimens in this study. Cost reduction was the only purpose for the optional screening assay, since we demonstrated that we could skip the screening assays for colorectal cancer specimens. Next, tumor-infiltrating T cells were stimulated with neoantigen-loaded DCs for 4 hours and subjected to a high-throughput single-cell sequencing analysis. To improve the sensitivity, the TCR and T-cell markers were target-enriched and then sequenced by an Illumina NextSeq sequencing system and an MiSeq sequencing system, respectively. The neoantigen-specific TCR sequences associated with T-cell activation markers, IFN-γ and IL-2, were identified by the bioinformatic analysis.

### Identifying neoantigen-specific TCRs directly from melanoma specimens

**Figure 2:**
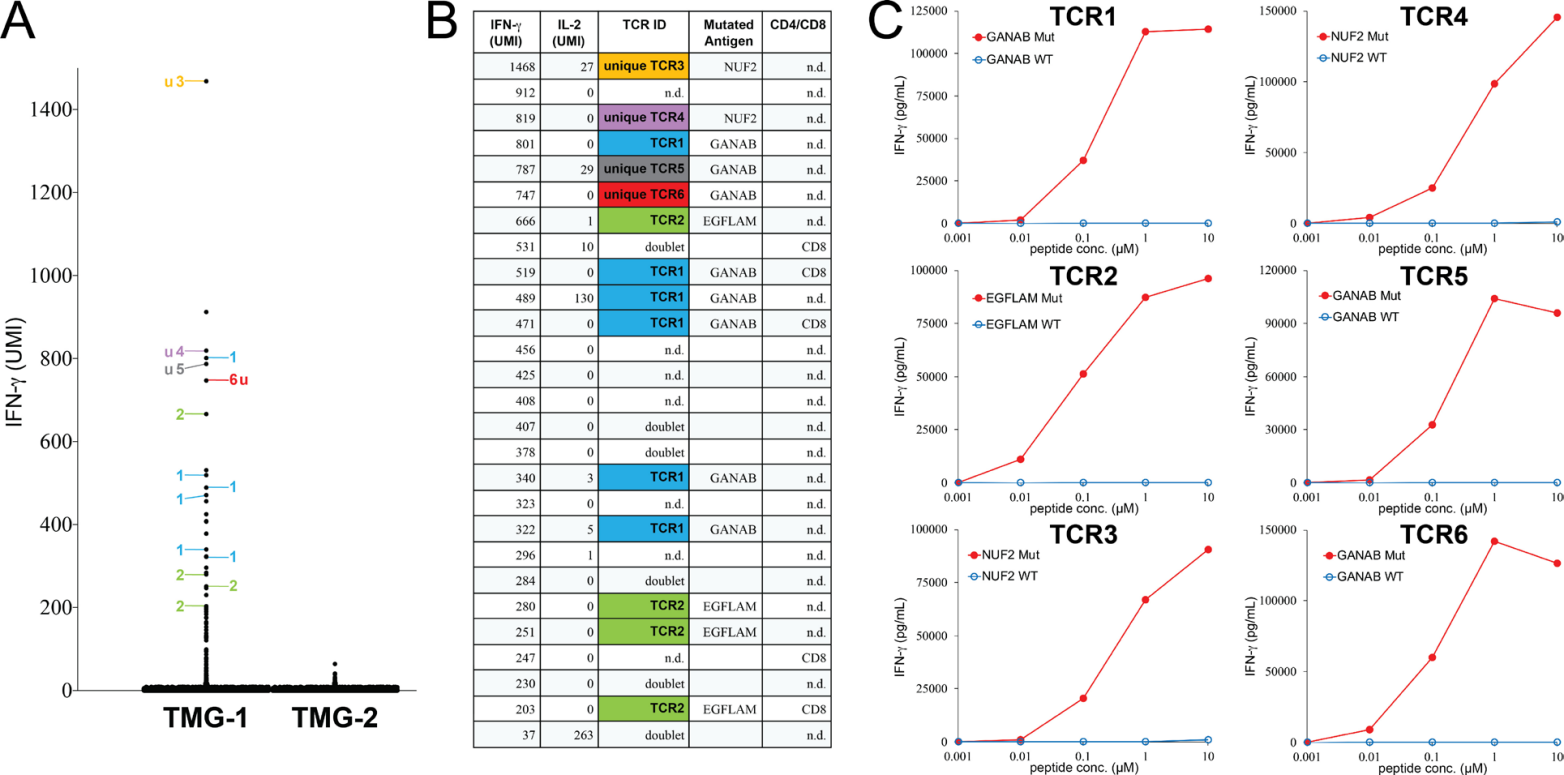
Isolation of neoantigen-specific TCRs from a metastatic tumor resected from patient M1 with melanoma. (A) T cells were stimulated by TMG-1 or TMG-2-transfected autologous DCs for 4 hours, and then subjected to single-cell analysis. The expression of IFN-γ in each single cell was obtained by bioinformatic analysis. The TCRs identified from individual single cells are labeled by numbers. Single cells with undetectable or mixed TCR sequences are not labeled. (B) The detailed information of each IFN-γ^+^ single cell is shown. n.d.: not detectable. (C) Individual TCRs isolated from tumor M1 were transduced into autologous T cells. Purified 25-mer WT or mutated peptide at various concentrations was pulsed on autologous DCs for 24 hours, and peptide-pulsed DCs were cocultured with TCR-transduced T cells for 16 hours. The secretion of IFN-γ from T cells was determined by ELISA. The gene names of neoantigen corresponding to individual neoantigen-specific TCRs are shown in each panel. DCs, dendritic cells; IFN-γ, interferon-γ; IL-2, interleukin-2; TCRs, T-cell receptors; u, unique TCR; UMI, unique molecular identifier; TMG, tandem minigene; WT, wild-type.

Initially, we attempted to isolate neoantigen-specific TCRs directly from melanoma specimens. A metastatic tumor was resected from patient M1 with advanced melanoma. The tumor specimen was cut into small pieces, dissociated into the single-cell suspension and then cryopreserved. The single-cell suspension was thawed and recovered overnight prior to the FACS sorting. To reduce the number of assays for the following single-cell analysis, sorted CD4^+^PD-1^+^ and CD8^+^PD-1^+^ T-cell populations were screened against autologous DCs expressing a TMG library encoding 64 non-synonymous mutations identified from the same tumor (online supplemental excel file sheet 1). The screening result indicated that a portion of T cells isolated from the tumor M1 single-cell suspension recognized TMG-1 (online supplemental figure 1A). Based on the positive reactivity, T cells isolated from the tumor M1 single-cell suspension were stimulated with DCs expressing TMG-1 for 4 hours, whereas TMG-2 was used as the negative control. Stimulated T cells were then subjected to the single-cell sequencing to obtain the expression levels of IFN-γ and IL-2, as well as TCR sequences, as described in the Methods section. As shown in figure 2A, a total of 26 single cells expressed high levels of IFN-γ after the stimulation by TMG-1. We analyzed the single-cell sequencing data and identified six different TCRs from those IFN-γ^+^ single cells (figure 2A). The IFN-γ levels and IL-2 levels, as well as the TCRs identified from these single-cells are listed in figure 2B. The TCR CDR3 region sequences are listed in online supplemental table 1).

10.1136/jitc-2021-002595.supp2 Supplementary data

10.1136/jitc-2021-002595.supp3 Supplementary data

To identify the specificities of these TCRs, full-length TCRs were synthesized and then transduced into autologous T cells isolated from the patient’s peripheral blood. TCR-transduced T cells were then cocultured with autologous DCs pulsed with 14 individual 25-mer mutated peptides corresponded to each minigene in TMG-1. Determined by IFN-γ ELISA assays, TCR1, TCR5 and TCR6 recognized mutated GANAB. TCR2 recognized mutated EGFLAM. TCR3 and TCR4 recognized mutated NUF2 (figure 2B). Furthermore, all six TCRs recognized (High-performance liquid chromatography) HPLC-purified, mutated 25-mer peptides, but not the wild-type (WT) counterpart (figure 2C).

Next, a metastatic tumor was resected from patient M2 with advanced melanoma. The tumor specimen was cut into small pieces, dissociated into a single cell suspension and then cryopreserved. The single cell suspension was thawed and recovered overnight prior to the FACS sorting. To reduce the number of samples for the following single-cell analysis, sorted CD4^+^PD-1^+^ and CD8^+^PD-1^+^ T-cell populations were screened against autologous DCs pulsed with PPs encoding 153 non-synonymous mutations identified from the same tumor (online supplemental excel file sheet 2). The screening results showed that T cells isolated from tumor M2 secreted higher levels of IFN-γ after coculture with autologous DCs pulsed with PP-1, PP-2 or PP-6 (online supplemental figure 1B).

**Figure 3:**
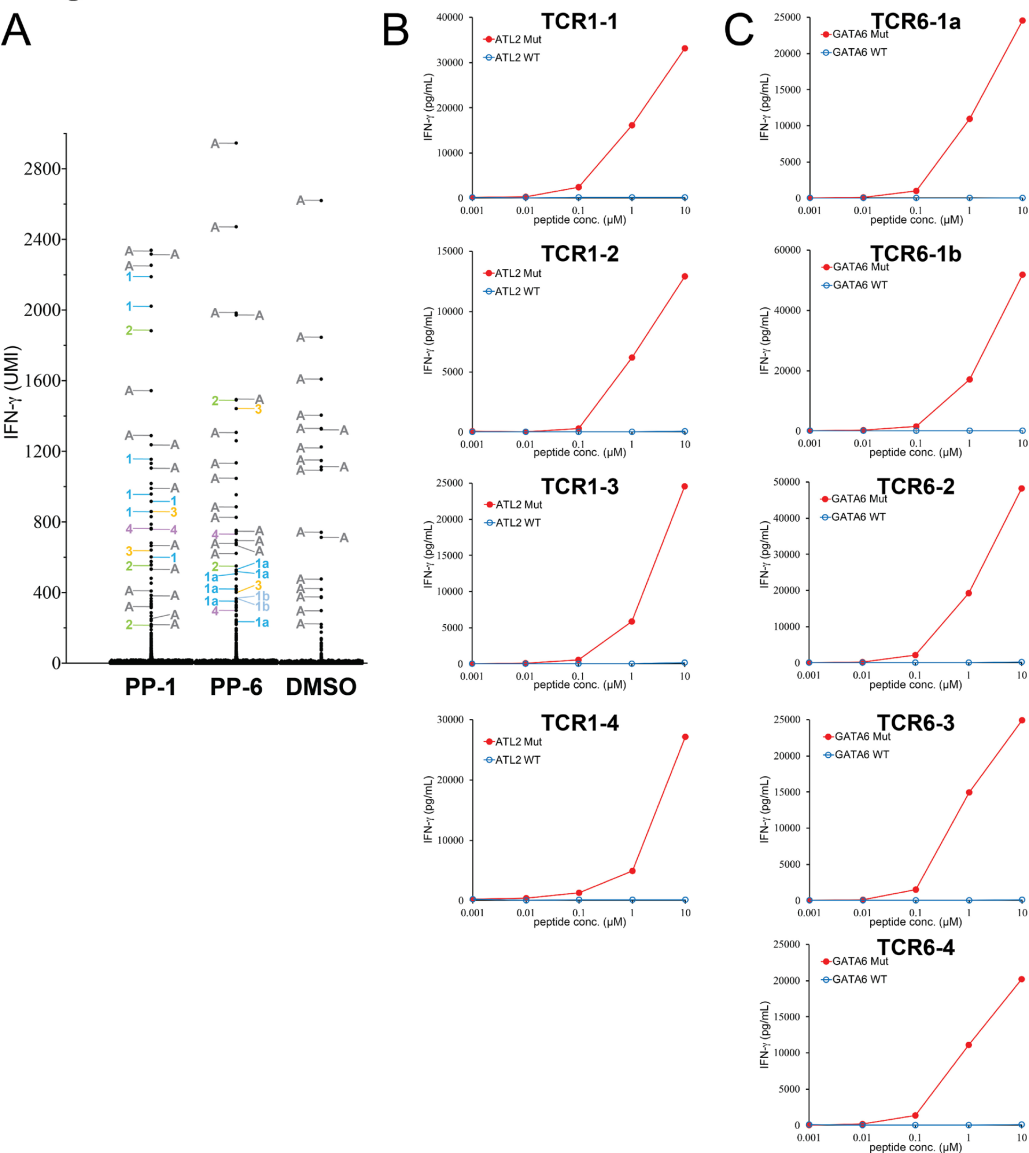
Identification of neoantigen-specific TCRs from a metastatic tumor resected from patient M2 with melanoma. (A) T cells were stimulated by PP-pulsed autologous DCs for 4 hours, and then subjected to single-cell analysis. The expression of IFN-γ in each single cell was obtained by bioinformatic analysis. The detailed information of each IFN-γ^+^ single cell is listed in online supplemental tables 2 and 3. The TCR identified from individual single cells are labeled by numbers (online supplemental tables 1-3). Unique TCRs are not labeled. (B, C) Individual TCRs isolated from tumor M2 were transduced into autologous T cells. Purified 25-mer WT or mutated peptide at various concentrations was pulsed on autologous DCs for 24 hours, and peptide-pulsed DCs were cocultured with TCR-transduced T cells for 16 hours. The secretion of IFN-γ from T cells was determined by ELISA. The gene names of neoantigen corresponding to individual neoantigen-specific TCRs are shown in each panel. A, autoreactive TCRs; DCs, dendritic cells; IFN-γ, interferon-γ; PPs, peptide pools; TCRs, T-cell receptors; UMI, unique molecular identifier; WT, wild-type.

Based on these results, T cells isolated from tumor M2 were stimulated with PP-1-, PP-2- or PP-6-pulsed DCs for 4 hours. Stimulated T cells were then subjected to the single-cell sequencing to obtain the expression levels of IFN-γ and IL-2, as well as TCR sequences. The same as the previous example, we analyzed single-cell TCR sequencing from IFN-γ^+^ populations. In this particular T-cell population, a subset of T cells with an autoreactive TCR (AV27/AV29/BV9) expressed high levels of IFN-γ after co-culture with autologous DCs without any peptide stimulation (DMSO negative control). These autoreactive T cells were not considered to be specific and removed from the analysis (online supplemental tables 1–3). In the PP-1 stimulated population, we identified four different TCRs (TCR1-1 to TCR1-4) shared by two or more single cells. In addition, five unique TCRs, not shared with other T cells, were also identified (figure 3A and online supplemental table 2). Because many TCRs were identified from this experiment, only TCRs (TCR1-1 to TCR1-4) shared by two or more single cells were studied in the subsequent experiments. In the PP-6 stimulated population, we identified five different TCRs (TCR6-1a to TCR6-4) shared by two or more single cells. Notably, TCR6-1a and TCR6-1b possessed nearly identical TCRα chain sequence, with the exception of a single silent nucleotide change at the CDR3A region. The β chains of TCR6-1a and TCR6-1b were similar, except two amino acid substitutions at the CDR3B region (online supplemental table 1). In addition, seven unique TCRs, not shared with other T cells, were also identified (figure 3A and online supplemental table 3). Because many TCRs were identified from this experiment, only TCRs (TCR6-1a to TCR6-4) shared by two or more single cells were studied in the subsequent experiments. In the PP-2 stimulated population, the majority of detectable TCRs within the IFN-γ^+^ population were autoreactive TCRs, and no additional shared TCRs were identified in this assay.

To identify the specificities of these TCRs, full-length TCRs were synthesized and then transduced into autologous T cells isolated from the patient’s peripheral blood. PPs PP-1 and PP-6 comprised 20 individual mutated peptides. TCR-transduced T cells were then cocultured with autologous DCs pulsed with individual 25-mer peptides from PP-1 or PP-6. Determined by IFN-γ ELISA assays, all four TCRs (TCR1-1 to TCR1-4) recognized mutated ALT2 from the PP-1 stimulated population (online supplemental table 2). From the PP-6 stimulated population, all five TCRs (TCR6-1a to TCR6-4) recognized mutated GATA6 (online supplemental table 3). Furthermore, all 11 two TCRs recognized HPLC-purified, mutated 25-mer peptides, but not the WT counterpart (figure 3B, C).

Lastly, a metastatic tumor was resected from patient M3 with advanced melanoma. To identify neoantigen-specific TCRs, T-cell populations from tumor M3 were sorted and screened against autologous DCs pulsed with PPs encoding 322 non-synonymous mutations identified from the same tumor (online supplemental excel file sheet 3). The screening results showed that T cells isolated from tumor M3 secreted higher levels of IFN-γ after coculture with autologous DCs pulsed with PP-1 (online supplemental figure 1C). Based on these results, T cells isolated from tumor M3 were stimulated with PP-1 pulsed DCs for 4 hours. Stimulated T cells were then subjected to the single-cell sequencing to obtain the expression levels of IFN-γ and IL-2, as well as TCR sequences. As for the previous example, we analyzed single-cell TCR sequencing from IFN-γ-high populations. In the PP-1 stimulated population, we identified three different TCRs (TCR1 to TCR3) shared by two or more single cells (online supplemental figure 2A). In addition, we also identified five unique TCRs (TCR4 to TCR8), which were not shared with other T cells. To identify the specificities of these TCRs, full-length TCRs were synthesized and then transduced into autologous T cells isolated from the patient’s peripheral blood. PP PP-1 comprised 20 individual mutated peptides (online supplemental excel file sheet 3). TCR-transduced T cells were then cocultured with autologous DCs pulsed with individual 25-mer peptides from PP-1. Determined by IFN-γ ELISA assays, TCR1 recognized mutated DDX39B. Additionally, TCR2, TCR3 and unique TCR4 recognized mutated RANBP2 (online supplemental table 4). However, unique TCR5 to TCR8 failed to recognize mutated peptides in PP-1. Lastly, TCR1 to TCR4 recognized HPLC-purified, mutated 25-mer peptides, but not the WT counterpart (online supplemental figure 2B).

### Isolating neoantigen-specific TCRs directly from colorectal tumor specimens

**Figure 4:**
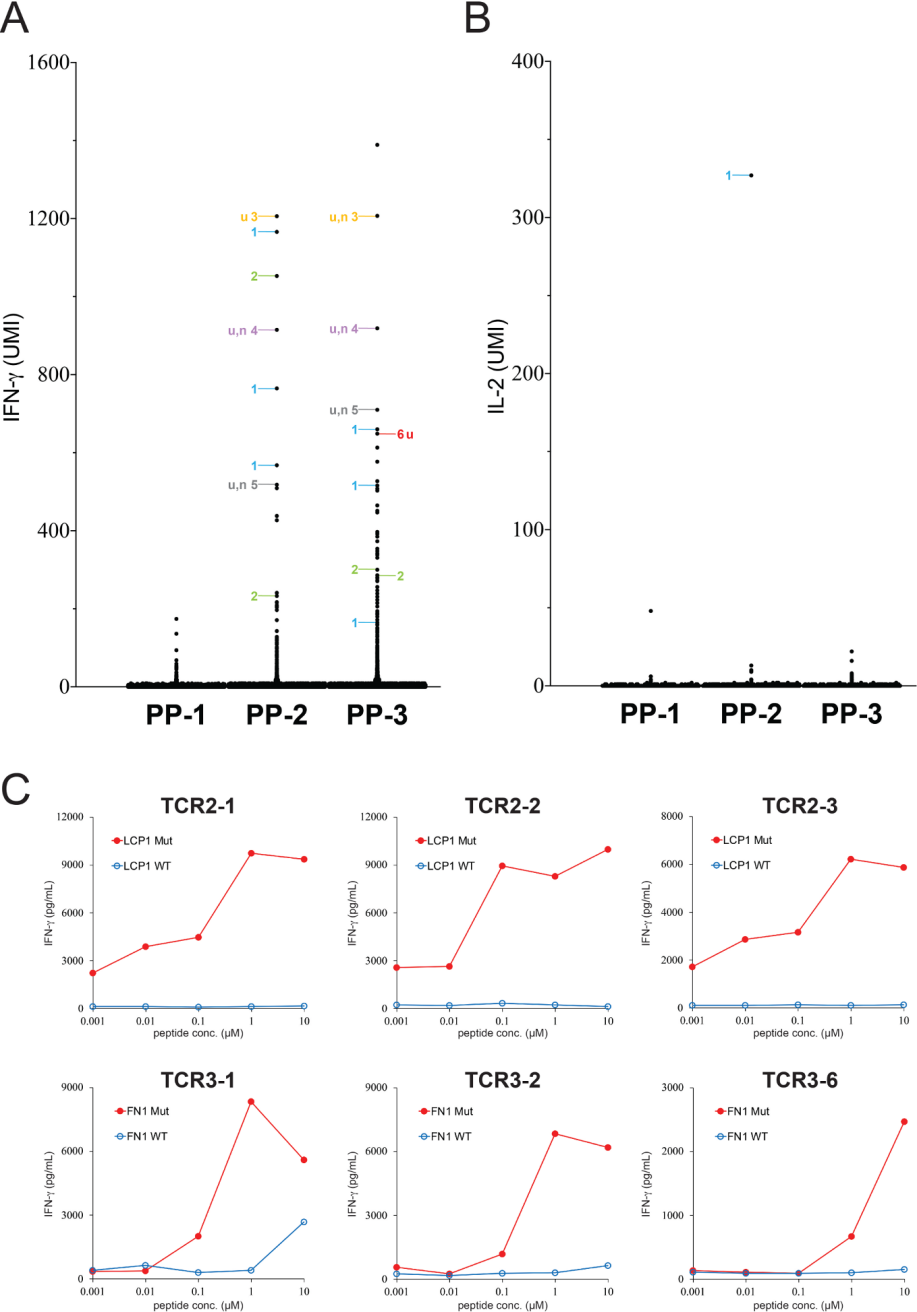
Isolation of neoantigen-specific TCRs from a metastatic tumor resected from patient CC1 with colorectal cancer. (A, B) Tumor-infiltrating T cells isolated from tumor CC1 were stimulated by PP-1, PP-2 or PP-3-pulsed autologous DCs for 4 hours, and then subjected to single-cell analysis. The expression of IFN-γ and IL-2 in each single cell was obtained by bioinformatic analysis. The detailed information of each IFN-γ^+^ and IL-2^+^ single cell is listed in online supplemental tables 5 and 6. The TCRs identified from individual single cells are labeled by numbers. Single cells with undetectable or mixed TCR sequences are not labeled. (C) Individual TCRs isolated from tumor CC1 were transduced into autologous T cells. HPLC-purified 25-mer WT or mutated peptide at various concentrations was pulsed on autologous DCs for 24 hours, and peptide-pulsed DCs were cocultured with TCR-transduced T cells for 16 hours. The secretion of IFN-γ from T cells was determined by ELISA. Only the reactive TCRs were shown. The gene names of neoantigen corresponding to individual neoantigen-specific TCRs are shown in each panel. DCs, dendritic cells; IFN-γ, interferon-γ; n, non-reactive TCR; TCRs, T-cell receptors; u, unique TCR; UMI, unique molecular identifier; WT, wild-type.

A metastatic tumor was resected from patient CC1 with colorectal cancer, and the tumor specimen was dissociated into a single cell suspension and cryopreserved. Because of the relatively low number of non-synonymous mutations identified from colorectal tumor specimens, we performed the single-cell analysis directly without the optional screening process. The single cell suspension was thawed and recovered overnight prior to the FACS sorting. After sorting, CD4^+^PD-1^+^ and CD8^+^PD-1^+^ T-cell populations were cultured in a medium containing the cytokine/antibody/inhibitor cocktail for 1 day, and then cultured in medium without the cocktail for 16 hours. T cells were stimulated by autologous DCs pulsed with three PPs (PP-1, PP-2 and PP-3) (online supplemental excel file sheet 4). The single-cell analysis showed that 17 single T cells isolated from tumor CC1 expressed high levels of IFN-γ after PP-2 stimulation, and 37 single T cells expressed high levels of IFN-γ after PP-3 stimulation (figure 4A). Notably, one T cell expressed a high level of IL-2 after PP-2 stimulation (figure 4B).

Following the same analysis as described previously, a total of 2 shared TCRs and 11 unique TCRs were identified after PP-2 stimulation (online supplemental table 5). Similarly, a total of 2 shared TCRs and 25 unique TCRs were identified after PP-3 stimulation (online supplemental table 6). Four shared TCRs were all tested, but only the top seven unique TCRs were tested. TCR2-1, TCR2-2 and unique TCR2-3 recognized mutated LCP1. In addition, TCR3-1, TCR3-2 and unique TCR3-6 recognized mutated FN1 (online supplemental tables 5 and 6). However, the rest of the unique TCRs did not recognize mutated peptides. Lastly, TCRs isolated from patient CC1 were tested against the HPLC-purified, mutated peptide, and the WT counterpart (figure 4C).

Lastly, tumors were resected from patient CC2 and CC3 with metastatic colorectal cancer. Sorted T-cell populations were stimulated by autologous DCs pulsed with PPs (online supplemental excel file sheet 5 and 6). The single-cell analysis showed that a shared TCR, CC2-TCR2-1, isolated from IFN-γ^+^ population of CC2 after PP-2 stimulation (online supplemental table 8). In addition, seven unique TCRs were isolated from CC2 and CC3 after the stimulation (online supplemental figures 3 and 4 and online supplemental tables 7–10). These TCRs were tested by following the same process. Shared CC2-TCR2-1 recognized mutated TNRC18. In addition, unique CC2-TCR2-2 recognized mutated RAPGEF1 (online supplemental figure 3), and unique CC3-TCR2-2 recognized mutated LRBA (online supplemental figure 4). However, the rest of the unique TCRs did not recognize any mutated peptides.

We observed highly variable levels of IFN-γ and IL-2 detected from individual single cells, as shown in figure 2A and online supplemental tables 2–10. Generally, IFN-γ^+^ single cells might or might not coexpress IL-2 in melanoma specimens. However, the vast majority of IL-2^+^ cells also coexpressed high levels of IFN-γ in melanoma specimens. On the contrary, IFN-γ and IL-2 did not coexpress in individual single cells isolated from colorectal cancer specimens. We need to study more tumor specimens to have a general conclusion about this observation, and it might be interesting to investigate the mechanism behind this.

### Identified neoantigen-specific TCR clonotypes represent small populations within the tumor-infiltrating T cells

**Figure 5:**
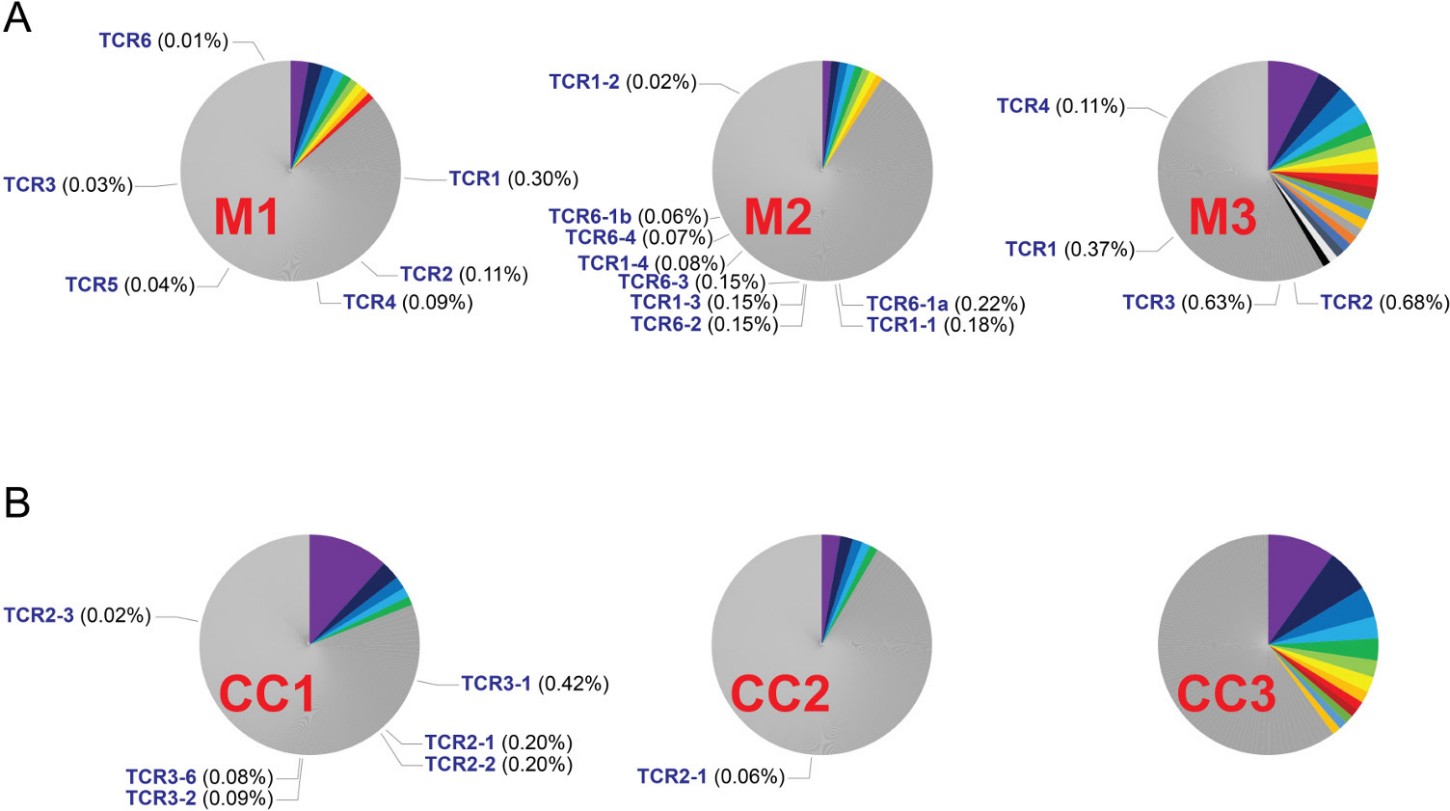
The frequencies of identified neoantigen-specific TCR clonotypes. Single-cell data generated from each tumor specimen were merged and analyzed. (A) The results of clonotype analysis for melanoma specimens are shown. (B) The results of clonotype analyses for colorectal cancer specimens are shown. TCR clonotypes with relatively high frequencies (>1%) were labeled with colors. TCR clonotypes that were only detected once were removed from the frequency calculation. The identified neoantigen-specific TCR clonotypes and their frequencies were indicated in each pie chart. Notably, the clonotypes of CC2 unique TCR2-2 and CC3 unique TCR2-2 were only detected once, and their frequencies were not calculated. TCRs, T-cell receptors

The TCR clonotypes in tumor-infiltrating T cells were highly diverse, and often very few T cells were detected in each clonotype in the single-cell analysis. To more accurately calculate the frequencies of identified TCR clonotypes, single-cell data generated from each tumor specimen were combined together and analyzed. In the experiment of tumor specimen M1, single-cell data from TMG-1 and TMG-2 stimulations were merged, and the TCR clonotypes with relatively high frequencies (>1%) were labeled with colors (figure 5A). The identified neoantigen-specific TCR clonotypes (TCR1 to TCR6) only represented a small portion of the entire population (frequency: 0.01%–0.30%). Although TCR1 is the top identified clonotype with the frequency at 0.30%, only 5 out of 15 single cells from TCR1 clonotype were IFN-γ^+^ after TMG-1 stimulation. In addition, four out of eight single cells from TCR2 clonotypes were IFN-γ^+^ after TMG-1 stimulation. The rest of the single cells were likely exhausted and failed to respond to the stimulation. However, it is difficult to generalize these findings due to low numbers of cells within each neoantigen-specific clonotypes.

Similar to tumor specimen M1, identified neoantigen-specific TCR clonotypes only represented small percentages of the T-cell populations (range: 0.02%–0.30%) (figure 5A and B). The total proportion of neoantigen-reactive TCRs in each tumor specimen was low in general (range: 0%–1.79%). Notably, the clonotypes of CC2 unique TCR2-2 and CC3 unique TCR2-2 were only detected once in the single-cell assays and their frequencies were not calculated. We excluded clonotypes that were detected once for the calculation of frequency, because many of these clonotypes appeared to be errors generated from doublets or sequencing errors.

In comparison, in our previous low-throughput single-cell approach (figure 1B), the frequencies of IFN-γ^+^, neoantigen-reactive T cells ranged from 2.1% to 23.0% in four tumor specimens. Notably, some clonotypes of neoantigen-reactive T cells could be significantly enriched during the long-term tumor fragment cultures. Based on the neoantigen reactivities, 1 of the 24 cultures was picked for the single-cell sequencing. This long process could greatly increase the proportions of some neoantigen-reactive T cells.

## Discussion

In this proof-of-concept study, we were able to identify neoantigen-specific TCRs from three melanoma specimens and three colorectal cancer specimens using this newly developed approach. This approach significantly reduced the time and labor compared with the previous approach using long-term TIL cultures. Notably, we failed to isolate neoantigen-specific TCRs from long-term TIL cultures from patients M3, CC1 and CC3. In addition, this approach could accurately identify neoantigen-specific TCRs, if identical TCR sequences were detected from two or more single cells (100%, 19 out of 19 shared TCRs). However, it was less accurate if TCR sequences were identified from only one single cell (39%, 9 out of 23 unique TCRs), likely due to sequencing errors or potential doublets. As a result, sequencing more single cells would likely obtain more identical TCRs, leading to more accurate results. One of the major limitations of this study was the cost associated with single-cell sample preparation and next-generation sequencing. Another major limitation was that a large number of tumor-infiltrating T cells were needed to perform the stimulation assay, followed by single-cell sequencing. Because the majority of the tumor specimens from these patients were used to generate long-term TIL cultures for treatments, the small amount of tumor specimens limited us to perform more comprehensive screening for the TCR identification using both TMG and peptide libraries.^14^

In recent years, several research groups have developed a variety of approaches to obtain TCR sequences. The most common approach involved sorting single T cells to individual wells in 96-well plates by FACS, based on the surface protein markers or tetramer staining. The TCR sequences from these single T cells were then obtained by PCR amplification and next-generation sequencing techniques.^36–38^ Notably, the construction of tetramer library requires either prior knowledge or prediction for minimum epitopes and human leuckocyte antigen (HLA) types. Except for several common HLAs, such as HLA-A*0201, the accuracy of minimum epitope prediction remains low. As a result, a large number of peptides are required for screening in order to obtain a positive hit.^38^ On the other side of the equation, several research groups develop new approaches to identify an unknown T-cell antigen recognized by a ‘known’ TCR with a ‘known’ DNA sequence.^39–42^ However, these approaches are extremely difficult to scale-up for hundreds of unknown TCRs with unknown specificities. For identifying unknown neoantigens, the TMG and peptide library screening remain the most efficient approach at this moment.^5^ Taken together, these recently developed approaches still require significant labor and time, not efficient enough to isolate neoantigen-specific TCRs. In comparison, the approach shown here is relatively fast and efficient.

The proportions of neoantigen and tumor-reactive TCRs were estimated by different approaches in several studies. Simoni *et al* utilized MHC-tetramer staining to screen putative neoantigen epitopes.^43^ Among 17 colorectal tumor specimens, two neoantigen-tetramer+ populations were identified from two specimens at 0.11% and 4.38% of the TILs, respectively. Notably, only one HLA type, A*11:01, was selected for tetramer screening in this study, except one patient. In another study, Scheper *et al* studied two ovarian and two colorectal cancer specimens and isolated about twenty TCRs from each specimen.^44^ These TCRs were transduced into healthy donor T cells and tested their reactivities against autologous tumor cells. Tumor-reactive TCRs were identified from one ovarian and one colorectal cancer specimen. The authors estimated that up to 10% of intratumoral CD8^+^ T cells could recognize autologous tumors. However, these TCRs could be isolated from exhausted T cells and regained their reactivities after expressing on healthy donor T cells. It is also unclear whether these tumor-reactive TCRs recognized neoantigens or non-mutated self-antigens. In our previous studies, long-term TIL fragment cultures were established from 10 metastatic gastrointestinal cancers.^45^ After TMG library screening, neoantigen-specific TCRs were isolated from neoantigen-reactive TIL cultures. TCRβ deep sequencing was performed on cryopreserved tumor specimens to track identified neoantigen-specific TCRs based on their CDR3 sequences. The frequencies of identified neoantigen-specific TCRs ranged from 0.009% to 1.3% among these tumors. In another study, top 10 most frequent TCRs from CD8^+^PD-1^+^ populations were isolated from 12 metastatic melanoma specimens.^46^ These TCRs were transduced into T cells and stimulated with neoantigens or autologous tumor cell lines. The frequencies of neoantigen-specific TCRs in tumor-infiltrating T-cell populations ranged from 0.18% to 7.25% in five specimens, and the frequencies of tumor-reactive TCRs ranged from 1.18% to 17.03% in 11 specimens.

Taken together, the frequencies of identified neoantigen-specific TCRs were low among the tumor-infiltrating T-cell populations, similar to the previous studies. Notably, PD-1 antibody (pembrolizumab) was added *in vitro* to prevent further exhaustion of T cells through the PD-1/PD-L1 pathway. However, it is possible that a proportion of neoantigen-specific T cells were totally exhausted, so they failed to respond to neoantigen stimulation. Additionally, some exhausted clonotypes might not be able to respond to stimulation at all, thus they might be difficult to be detected by this approach. Therefore, the observation from this study might imply that only a small portion of T cells are capable of killing tumor cells in the tumor microenvironment. The vast majority of tumor-infiltrating T cells are either exhausted T cells or bystanders. Because we have only studied a limited numbers of tumor specimens, a large-scale study is required to test this hypothesis.

In summary, we have developed a new approach to quickly identify neoantigen-specific TCRs directly from tumor specimens. In this study, PD-1 marker was used to enrich neoantigen-reactive T cells from tumor specimens.^27–29^ However, recent results suggested that PD-1 might not be the best marker for neoantigen-reactive T cells.^43^ Other markers, such as CD39 and CD103, have been proposed as more precise markers for neoantigen-reactive T cells. These markers will be tested in the future.^43 47–49^ Lastly, thanks to the intensive research on single-cell genomics in recent years, the quality, sensitivity and cost of single-cell sequencing technique have been improved dramatically, and likely so in the coming years. As a result, the efficiency of this approach will likely be improved in the near future as well.

## Data Availability

Data are available upon reasonable request. Next-generation sequencing data will be available upon request, following publication of this manuscript.
