# Supplementary material for: Direct identification of neoantigen-specific TCRs from tumor specimens by high-throughput single-cell sequencing
Source: J Immunother Cancer. 2021 Jul 28;9(7):e002595. doi: 10.1136/jitc-2021-002595 (PMC8320258; doi:10.1136/jitc-2021-002595)
Supplement: Supplementary data [file jitc-2021-002595supp002.pdf]

Supplementary Figures

Title:

Direct identification of neoantigen-specific TCRs from tumor specimens by high-throughput single-cell sequencing

Authors:

Yong-Chen Lu[1, 2], Zhili Zheng[1], Frank J. Lowery[1], Jared J. Gartner[1], Todd D. Prickett[1], Paul F. Robbins[1], Steven A. Rosenberg[1]

[1]Surgery Branch, National Cancer Institute, National Institutes of Health, Bethesda, MD 20892, USA.

[2]Department of Pathology and Winthrop P. Rockefeller Cancer Institute, University of Arkansas for Medical Sciences, Little Rock, Arkansas, USA.

Lu Y-C, *et al. J Immunother Cancer* 2021; 9:e002595. doi: 10.1136/jitc-2021-002595

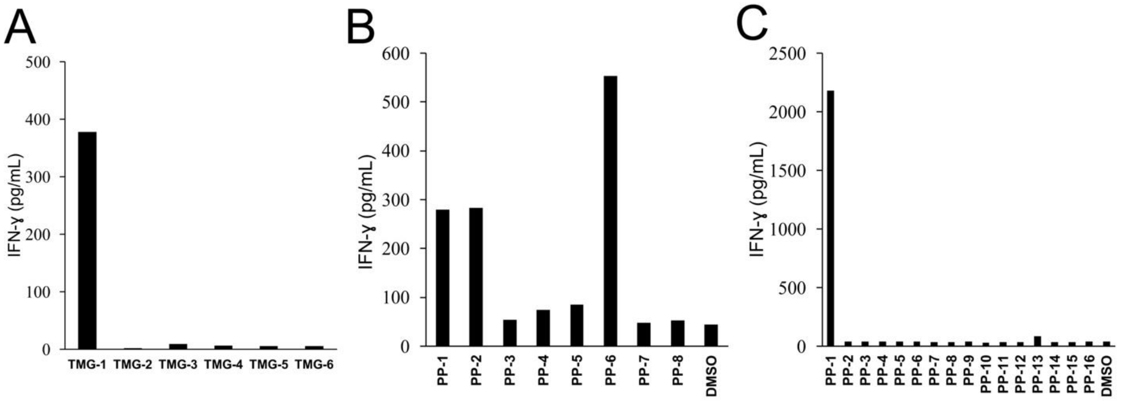

Supplementary Figure 1. Screening assays for melanoma specimens. **(A)** Tumor-infiltrating T cells isolated from tumor M1 were screened against a tandem minigene (TMG) library encoding non-synonymous mutations identified from tumor M1. The reactivities of T cells against TMGs were measured by the levels of IFN-γ secreted by T cells. **(B)** Tumor-infiltrating T cells isolated from tumor M2 were screened against a peptide pool (PP) library encoding non-synonymous mutations identified from tumor M2. The reactivities of T cells against PPs were determined by ELISA. **(C)** Tumor-infiltrating T cells isolated from tumor M3 were screened against a PP library encoding non-synonymous mutations identified from tumor M3. The reactivities of T cells against PPs were determined by ELISA.

Lu Y-C, *et al. J Immunother Cancer* 2021; 9:e002595. doi: 10.1136/jitc-2021-002595

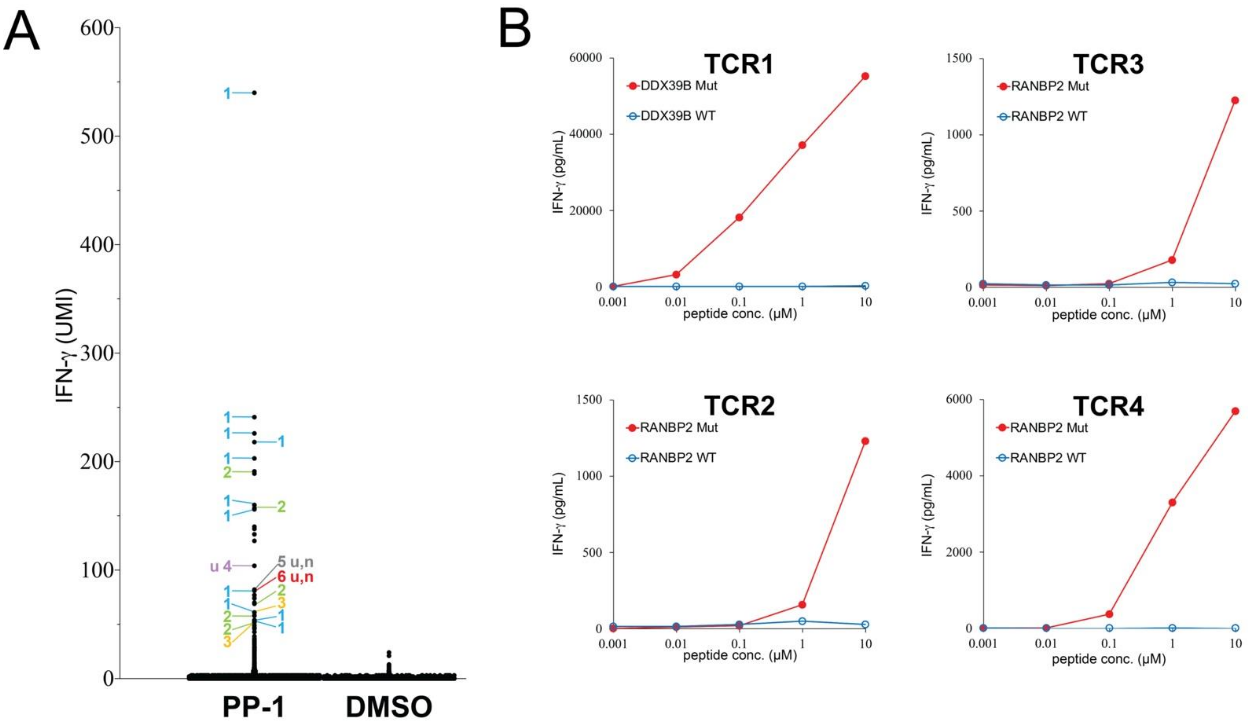

Supplementary Figure 2. Isolation of neoantigen-specific TCRs from a metastatic tumor resected from patient M3 with metastatic melanoma. **(A)** Tumor-infiltrating T cells isolated from tumor M3 were screened against a peptide library encoding non-synonymous mutations identified from tumor M3. The reactivity of T cells against peptide pools (PP) was measured by IFN-γ ELISA. **(B)** T cells were stimulated by PP-pulsed autologous DCs for 4 hr, and then subjected to single-cell analysis. The expression of IFN-γ in each single cell was obtained by bioinformatics analysis. The detailed information of each IFN-γ^{+} single cell is listed in Supplementary Table 4. The TCR identified from individual single cells are labeled by numbers (Supplementary Table 1 and 4). UMI: unique molecular identifier; u: unique TCR; n: non-reactive TCR. **(C)** Individual TCRs isolated from tumor M3 was transduced into autologous T cells. Purified 25-mer WT or mutated peptide at various concentrations was pulsed on autologous DCs for 24 hr, and peptide-pulsed DCs were co-cultured with TCR-transduced T cells for 16 hr. The secretion of IFN-γ from T cells was determined by ELISA. Only the reactive TCRs were shown. The gene names of neoantigen corresponding to individual neoantigen-specific TCRs are shown in each panel.

Lu Y-C, *et al. J Immunother Cancer* 2021; 9:e002595. doi: 10.1136/jitc-2021-002595

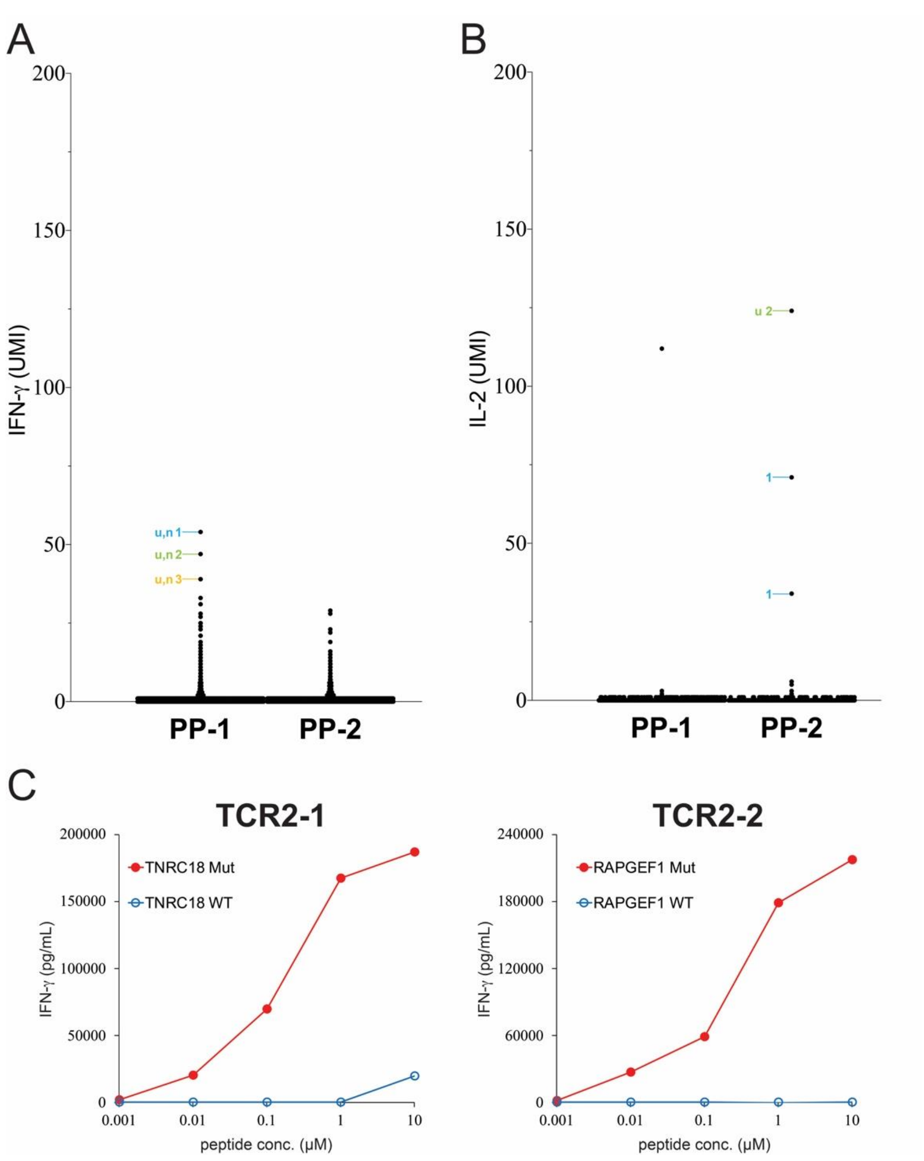

Supplementary Figure 3. Isolation of neoantigen-specific TCRs from a metastatic tumor resected from patient CC2 with colorectal cancer. **(A) (B)** Tumor-infiltrating T cells were stimulated by PP-pulsed autologous DCs for 4 hr, and then subjected to single-cell analysis. The expression of IFN-γ and IL-2 in each single cell was shown. The detailed information of each IFN-γ^+ or IL-2^+ single cell is listed in Supplementary Table 7 and 8. The TCR identified from individual single cells are labeled by numbers (Supplementary Table 1, 7 and 8). UMI: unique molecular identifier; u: unique TCR; n: non-reactive TCR. **(C)** Individual TCRs isolated from tumor CC2 was transduced into autologous T cells. HPLC-purified 25-mer WT or mutated peptide at various concentrations was pulsed on autologous DCs for 24 hr, and peptide-pulsed DCs were co-cultured with TCR-transduced T cells for 16 hr. The secretion of IFN-γ from T cells was determined by ELISA. Only the reactive TCRs were shown. The gene names of neoantigen corresponding to individual neoantigen-specific TCRs are shown in each panel.

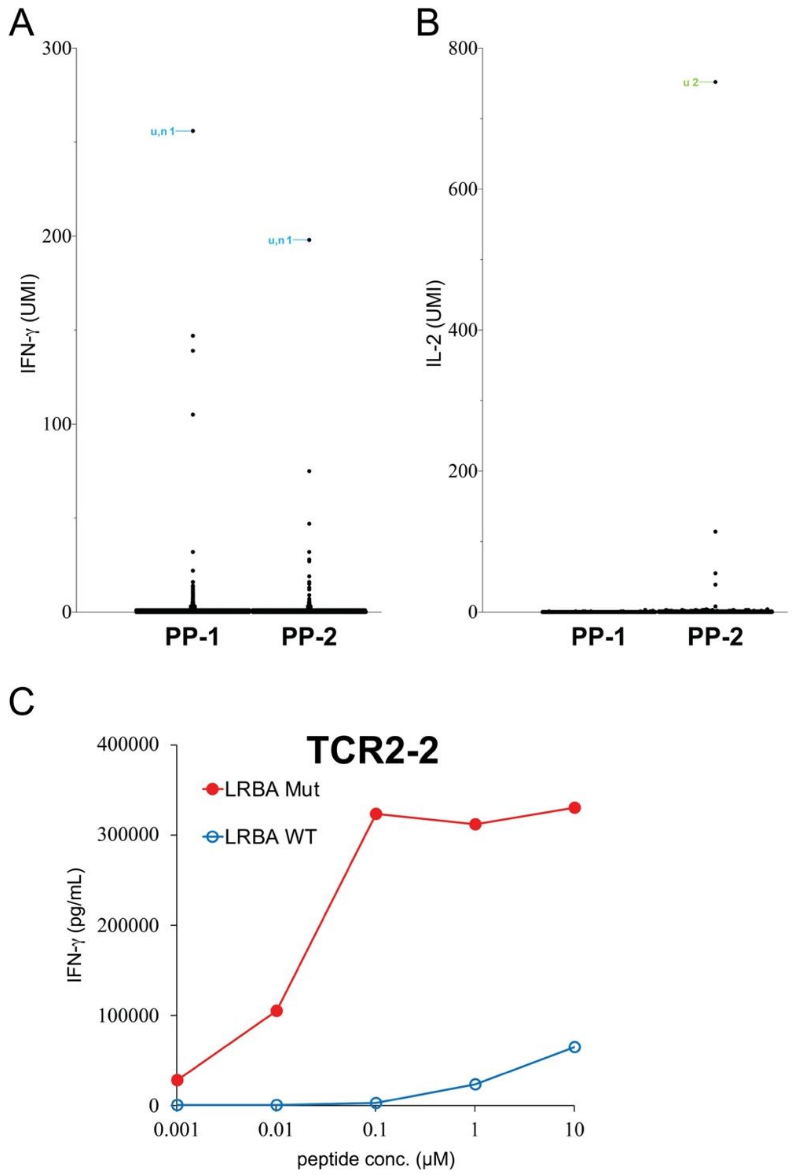

Supplementary Figure 4. Identification of a neoantigen-specific TCR from a metastatic tumor resected from patient CC3 with colorectal cancer. **(A) (B)** Tumor-infiltrating T cells isolated from tumor CC3 T cells were stimulated by PP-pulsed autologous DCs for 4 hr, and then subjected to single-cell analysis. The expression of IFN-γ **(A)** and IL-2 **(B)** in each single cell was obtained by bioinformatics analysis. The detailed information of each IFN-γ^+ and/or IL-2^+ single cell is listed in Supplementary Table 9 and 10. The TCR identified from individual single cells are labeled by numbers (Supplementary Table 1, 9 and 10). UMI: unique molecular identifier; u: unique TCR; n: non-reactive TCR. **(C)** Individual TCRs isolated from tumor CC3 was transduced into donor T cells. Autologous DCs were pulsed with purified 25-mer mutated LRBA peptide or the corresponding WT peptide at various concentrations for 24 hr, and peptide-pulsed DCs were co-cultured with transduced T cells for 16 hr. The secretion of IFN-γ from T cells was determined by ELISA. Only the reactive TCR was shown.

Lu Y-C, *et al. J Immunother Cancer* 2021; 9:e002595. doi: 10.1136/jitc-2021-002595
