# Supplementary material for: Direct identification of neoantigen-specific TCRs from tumor specimens by high-throughput single-cell sequencing
Source: J Immunother Cancer. 2021 Jul 28;9(7):e002595. doi: 10.1136/jitc-2021-002595 (PMC8320258; doi:10.1136/jitc-2021-002595)
Supplement: Supplementary data [file jitc-2021-002595supp003.pdf]

Supplementary Tables

Title:

Direct identification of neoantigen-specific TCRs from tumor specimens by high-throughput single-cell sequencing

Authors:

Yong-Chen Lu[1, 2], Zhili Zheng[1], Frank J. Lowery[1], Jared J. Gartner[1], Todd D. Prickett[1], Paul F. Robbins[1], Steven A. Rosenberg[1]

[1]Surgery Branch, National Cancer Institute, National Institutes of Health, Bethesda, MD 20892, USA.

[2]Department of Pathology and Winthrop P. Rockefeller Cancer Institute, University of Arkansas for Medical Sciences, Little Rock, Arkansas, USA.

Supplementary Table 1. The summary of TCR and CDR3 information

Patient ID	TCR ID	AV	CDR3A	BV	CDR3B	Mutated Antigen
M1	TCR1	19	CALRGGDSNYQLIW	24-1	CATSDSGPGEQYF	GANAB
M1	TCR2	8-4	CAVSDRNNDMRF	13	CASSLVPRARGTEAFF	EGFLAM
M1	unique TCR3	19	CALSERYTGTASKLTF	4-1	CASSQERQTSYEQYF	NUF2
M1	unique TCR4	8-3	CAVGARFSSNTGKLIF	7-3	CASSPGSTSGRVRETQYF	NUF2
M1	unique TCR5	19	CALSGWNRDDKIIF	12-4	CASSGGATYNEQFF	GANAB
M1	unique TCR6	8-3	CATINFGNEKLTF	5-1	CASSLDQGLNTEAFF	GANAB
M2	TCR1-1	25	CAGTPSGSARQLTF	7-6	CASAGTSGWQETQYF	ATL2
M2	TCR1-2	35	CAGHGGATNKLIF	7-6	CASSLAPQGEAFF	ATL2
M2	TCR1-3	24	CASYTNAGKSTF	4-2	CASSQERGLAGFNEQFF	ATL2
M2	TCR1-4	35	CAGQLISGAGSYQLTF	4-1	CASSQALAGPEQFF	ATL2
M2	TCR6-1a	12-3	CAMSSYSSASKIIF	12-4	CASSFEADTQYF	GATA6
M2	TCR6-1b	12-3	CAMSSYSSASKIIF	12-4	CATSLEADTQYF	GATA6
M2	TCR6-2	26-2	CILSGGGSNYKLTF	12-4	CASSLYGPLSSYNEQFF	GATA6
M2	TCR6-3	26-2	CILRENNARLMF	12-4	CASSLEATSVYNEQFF	GATA6
M2	TCR6-4	38-1	CASRTDSWGKLQF	19	CASSIDGAGGPPGELFF	GATA6
M2	Auto-reactive (A)	27/ 29	CAAMNLSSNTGKLIF/ CAASAYEVHDMRF	9	CASSAFQGLPLSGNTIYF	negative
M3	TCR1	2	CAVEEIQAGTALIF	2	CASSTQGLYGYTF	DDX39B
M3	TCR2	12-1	CVVKGSGYSSASKIIF	10-2	CASTQGGGLAPHYEQYF	RANBP2
M3	TCR3	4	CLTSGAGSYQLTF	28	CASSSRTSGRHNEQFF	RANBP2
M3	unique TCR4	26-1	CIVTGYSSASKIIF	24-1	CATSDFRAAWYEQYF	RANBP2
M3	unique TCR5	14	CAMRESANQAGTALIF	25-1	CASSDTGQGLIGELFF	negative
M3	unique TCR6	5	CAEIGGSNYKLTF	25-1	CASSDLGAPEQYF	negative
M3	unique TCR7	10	CVVTRGSNYQLIW	7-3	CASAPGGYTTDTQYF	negative
M3	unique TCR8	12-3	CAMRGNYGQNFVF	20-1	CSAREAGGGGNTIYF	negative
CC1	TCR2-1	8-1	CAVNGNKLVF	15	CATSREGQDTHLGEQYF	LCP1
CC1	TCR2-2	6	CALEPMDSSYKLIF	7-9	CASSLTGETQYF	LCP1
CC1	unique TCR2-3	9-2	CAVLTGANNLFF	7-2	CASSLAGQSSYEQYF	LCP1
CC1	unique TCR2-4	2	CAVVGDDKIIF	6-6	CASRATGGVSNQPQHF	negative
CC1	unique TCR2-5	13-1	CAARPPGAGSYQLTF	6-3	CASSLLAGGNEQFF	negative
CC1	TCR3-1	27	CAGLNAGNNRKLIW	7-9	CASSLGSAKATNEKLFF	FN1
CC1	TCR3-2	8-6/ 14*	CAVSPTGSARQLTF / CAMREGGYNFNKFYF*	6-3	CASRGAPGRENTEAFF	FN1
CC1	unique TCR3-3	20	CAAVTTDSWGKLQF	29-1	CSVGSAGSDTQYF	negative
CC1	unique TCR3-4	14	CAMREVDIGGFKTIF	2	CASSVLVSSGNTIYF	negative
CC1	unique TCR3-5	8-4	CAVSRGQAGTALIF	25-1	CASSEWGGPYEQYF	negative
CC1	unique TCR3-6	22	CAVARGGKLIF	11-2	CASSLEAGSTDTQYF	FN1
CC2	unique TCR1-1	21	CAVKLSNYQLIW	5-1	CASSSSDTGYTF	negative
CC2	unique TCR1-2	26-1	CIVRVALDRNNNARLMF	6-5	CASSYGTAYGYTF	negative

CC2	unique TCR1-3	12-3	CAMRRASGGSYIPTF	6-5	CASSYREGLYEQYF	negative
CC2	TCR2-1	17/<br>23*	CATDAGPGTASKLTF /<br>CAVDSGGGADGLTF*	20-1	CSARKTGRYEQYF	TNRC18
CC2	unique TCR2-2	21	CAVSLTGGGNKLTF	20-1	CSASRGETQYF	RAPGEF1
CC3	unique TCR1-1	41	CAVRIIQGAQKLVF	7-8	CASSQGVWGAGNTIYF	negative
CC3	unique TCR2-1	13-1<br>26-1	CAAKDSGGGADGLTF<br>CIVYAGEYGNKLVF	7-9	CASSPDAANYGYTF	negative
CC3	unique TCR2-2	17	CATLSPTTSGTYKYIF	11-1	CASSLGTSGLYNEQFF	LRBA

*After pairing with the TCRβ chains identified from the same single cells, these TCRα chains did not recognize the mutated peptides.

Supplementary Table 2. M2-TCR identification after PP-1 stimulation

IFN-γ (UMI)	IL-2 (UMI)	TCR ID	Mutated Antigen	CD4/CD8
2339	60	autoreactive TCR		n.d.
2317	17	autoreactive TCR		n.d.
2254	225	autoreactive TCR		n.d.
2189	9	TCR1-1	ATL2	n.d.
2022	112	TCR1-1	ATL2	CD8
1883	23	TCR1-2	ATL2	CD8
1544	30	autoreactive TCR		n.d.
1289	0	autoreactive TCR		n.d.
1237	90	autoreactive TCR		n.d.
1155	1	TCR1-1	ATL2	n.d.
1132	11	not detectable		n.d.
1105	2	autoreactive TCR		CD8
1018	24	unique TCR		CD8
990	9	autoreactive TCR		CD8
958	10	TCR1-1	ATL2	n.d.
917	11	TCR1-1	ATL2	n.d.
859	0	TCR1-3	ATL2	n.d.
859	88	TCR1-1	ATL2	CD8
830	228	autoreactive TCR		n.d.
787	0	autoreactive TCR		n.d.
764	0	TCR1-4	ATL2	CD8
760	1	TCR1-4	ATL2	n.d.
681	0	doublet		CD8
665	0	autoreactive TCR		n.d.
641	0	TCR1-3	ATL2	n.d.
602	0	TCR1-1	ATL2	CD8
576	10	autoreactive TCR		n.d.
576	37	not detectable		n.d.
558	0	autoreactive TCR		n.d.
556	0	TCR1-2	ATL2	CD8
534	0	autoreactive TCR		n.d.
532	1	unique TCR		CD8
481	0	unique TCR		CD8
453	2	not detectable		n.d.
409	1	autoreactive TCR		n.d.
385	2	autoreactive TCR		n.d.
384	1	not detectable		n.d.
366	27	not detectable		n.d.
352	6	autoreactive TCR		n.d.
339	0	autoreactive TCR		n.d.
331	0	not detectable		CD8
331	11	unique TCR		n.d.
324	0	autoreactive TCR		n.d.
320	0	autoreactive TCR		n.d.
315	34	not detectable		CD8
286	0	not detectable		n.d.
268	0	doublet		mixed
250	0	autoreactive TCR		n.d.
241	1	not detectable		n.d.
220	1	autoreactive TCR		n.d.
217	0	TCR1-2	ATL2	CD8
215	0	doublet		CD8
208	0	unique TCR		n.d.

Supplementary Table 3. M2-TCR identification after PP-6 stimulation

IFN-γ (UMI)	IL-2 (UMI)	TCR ID	Mutated Antigen	CD4/CD8
2946	355	autoreactive TCR		n.d.
2472	1	autoreactive TCR		CD8
1983	22	autoreactive TCR		n.d.
1972	64	autoreactive TCR		mixed
1493	24	autoreactive TCR		n.d.
1491	13	TCR6-2	GATA6	CD8
1442	2	TCR6-3	GATA6	CD8
1307	84	autoreactive TCR		n.d.
1260	0	doublet		mixed
1135	2	autoreactive TCR		n.d.
1047	145	autoreactive TCR		n.d.
954	1	unique TCR		CD8
886	1	autoreactive TCR		CD8
826	51	autoreactive TCR		n.d.
753	0	unique TCR		CD8
746	0	autoreactive TCR		n.d.
735	0	TCR6-4	GATA6	CD8
696	35	autoreactive TCR		n.d.
681	52	autoreactive TCR		n.d.
672	1	autoreactive TCR		n.d.
622	1	autoreactive TCR		n.d.
551	2	TCR6-2	GATA6	CD8
528	1	TCR6-1a	GATA6	CD8
521	1	TCR6-1a	GATA6	CD8
507	1	TCR6-1a	GATA6	CD8
477	0	unique TCR		CD8
477	14	autoreactive TCR		n.d.
436	0	not detectable		n.d.
422	0	TCR6-1a	GATA6	CD8
416	0	doublet		n.d.
414	4	unique TCR		CD8
402	0	TCR6-3	GATA6	CD8
365	0	TCR6-1b	GATA6	CD8
365	6	TCR6-1b	GATA6	CD8
352	0	TCR6-1a	GATA6	CD8
346	0	unique TCR		CD8
329	0	not detectable		CD8
318	6	unique TCR		n.d.
318	0	autoreactive		n.d.
314	10	autoreactive		n.d.
299	0	unique TCR		n.d.
299	34	TCR6-4	GATA6	CD8
275	12	autoreactive TCR		mixed
274	7	not detectable		CD8
270	0	autoreactive TCR		n.d.
268	2	unique TCR		CD8
245	34	autoreactive TCR		n.d.
240	0	autoreactive TCR		mixed
232	1	TCR6-1a	GATA6	CD8
223	0	autoreactive TCR		CD8
220	3	unique TCR		CD8

Lu Y-C, *et al. J Immunother Cancer* 2021; 9:e002595. doi: 10.1136/jitc-2021-002595

Supplementary Table 4. M3-TCR identification after PP-1 stimulation

IFN-γ (UMI)	IL-2 (UMI)	TCR ID	Mutated Antigen	CD4/CD8
540	84	TCR1	DDX39B	n.d.
241	23	TCR1	DDX39B	CD8
226	10	TCR1	DDX39B	n.d.
218	8	TCR1	DDX39B	CD8
203	15	TCR1	DDX39B	n.d.
191	1	TCR2	RANBP2	CD8
189	25	not detectable		n.d.
160	11	TCR1	DDX39B	n.d.
157	3	TCR2	RANBP2	n.d.
156	10	TCR1	DDX39B	CD8
140	0	not detectable		n.d.
138	54	not detectable		n.d.
133	59	not detectable		n.d.
127	1	not detectable		n.d.
104	6	unique TCR4	RANBP2	n.d.
82	21	not detectable		n.d.
81	1	unique TCR5	negative	CD8
77	0	TCR1	DDX39B	n.d.
77	0	unique TCR6	negative	CD8
74	52	doublet		n.d.
74	3	not detectable		n.d.
70	12	not detectable		n.d.
69	1	TCR2	RANBP2	n.d.
61	1	TCR3	RANBP2	CD8
61	5	TCR1	DDX39B	n.d.
58	0	unique TCR7	negative	CD8
58	42	doublet		n.d.
58	0	TCR2	RANBP2	n.d.
54	18	TCR1	DDX39B	n.d.
53	56	TCR1	DDX39B	n.d.
53	6	TCR2	RANBP2	n.d.
52	0	TCR3	RANBP2	CD8
52	0	unique TCR8	negative	CD8

Lu Y-C, *et al. J Immunother Cancer* 2021; 9:e002595. doi: 10.1136/jitc-2021-002595

Supplementary Table 5. CC1-TCR identification after PP-2 stimulation

IFN-γ (UMI)	IL-2 (UMI)	TCR ID	Mutated Antigen	CD4/CD8
1206	0	unique TCR2-3	LCP1	n.d.
1166	0	TCR2-1	LCP1	n.d.
1053	0	TCR2-2	LCP1	n.d.
915	0	unique TCR2-4	negative	n.d.
765	3	TCR2-1	LCP1	n.d.
568	0	TCR2-1	LCP1	n.d.
518	0	unique TCR2-5	negative	CD8
509	0	unique TCR	not tested	CD8
438	2	unique TCR	not tested	n.d.
427	0	unique TCR	not tested	n.d.
241	0	unique TCR	not tested	n.d.
233	0	TCR2-2	LCP1	n.d.
217	0	unique TCR	not tested	n.d.
216	0	unique TCR	not tested	n.d.
209	0	doublet		CD8
206	0	unique TCR	not tested	n.d.
204	0	unique TCR	not tested	CD8
36	327	TCR2-1	LCP1	n.d.

Lu Y-C, *et al. J Immunother Cancer* 2021; 9:e002595. doi: 10.1136/jitc-2021-002595

Supplementary Table 6. CC1-TCR identification after PP-3 stimulation

IFN-γ (UMI)	IL-2 (UMI)	TCR ID	Mutated Antigen	CD4/CD8
1389	0	doublet		CD8
1207	0	unique TCR3-3	negative	n.d.
919	0	unique TCR3-4	negative	CD8
710	0	unique TCR3-5	negative	n.d.
660	0	TCR3-1	FN1	n.d.
649	0	unique TCR3-6	FN1	n.d.
613	0	unique TCR	not tested	n.d.
577	0	unique TCR	not tested	n.d.
527	0	unique TCR	not tested	n.d.
516	0	TCR3-1	FN1	n.d.
508	0	unique TCR	not tested	n.d.
503	0	unique TCR	not tested	n.d.
465	0	unique TCR	not tested	n.d.
451	0	Doublet		CD8
447	0	unique TCR	not tested	n.d.
397	16	not detectable		n.d.
392	0	unique TCR	not tested	CD8
385	0	unique TCR	not tested	n.d.
373	0	unique TCR	not tested	n.d.
354	0	unique TCR	not tested	CD8
348	0	Doublet		CD8
342	0	unique TCR	not tested	n.d.
338	0	not detectable		n.d.
331	0	unique TCR	not tested	CD8
300	0	TCR3-2	FN1	n.d.
286	0	TCR3-2	FN1	n.d.
280	0	unique TCR	not tested	n.d.
279	0	unique TCR	not tested	n.d.
273	0	unique TCR	not tested	n.d.
271	0	not detectable		n.d.
256	0	unique TCR	not tested	CD8
247	0	unique TCR	not tested	n.d.
239	0	doublet		n.d.
231	0	unique TCR	not tested	CD8
223	0	unique TCR	not tested	CD8
215	0	TCR3-1	FN1	mixed
206	0	unique TCR	not tested	CD8

Supplementary Table 7. CC2-TCR identification after PP-1 stimulation

IFN-γ (UMI)	IL-2 (UMI)	TCR ID	Mutated Antigen	CD4/CD8
54	0	unique TCR1-1	negative	CD8
47	0	unique TCR1-2	negative	CD8
39	0	unique TCR1-3	negative	CD8
39	0	doublet		CD8
0	112	not detectable		n.d.

Supplementary Table 8. CC2-TCR identification after PP-2 stimulation

IFN-γ (UMI)	IL-2 (UMI)	TCR ID	Mutated Antigen	CD4/CD8
0	124	unique TCR2-2	RAPGEF1	mixed
1	71	TCR2-1	TNRC18	CD8
1	34	TCR2-1	TNRC18	CD8

Supplementary Table 9. CC3-TCR identification after PP-1 stimulation

IFN-γ (UMI)	IL-2 (UMI)	TCR ID	Mutated Antigen	CD4/CD8
256	0	unique TCR1-1	negative	CD8
147	0	not detectable		n.d.
139	0	not detectable		CD8
105	0	not detectable		n.d.

Supplementary Table 10. CC3-TCR identification after PP-2 stimulation

IFN-γ (UMI)	IL-2 (UMI)	TCR ID	Mutated Antigen	CD4/CD8
198	0	unique TCR2-1	negative	n.d.
75	0	not detectable		n.d.
28	752	unique TCR2-2	LRBA	n.d.
3	114	not detectable		n.d.
3	55	not detectable		n.d.

Lu Y-C, *et al. J Immunother Cancer* 2021; 9:e002595. doi: 10.1136/jitc-2021-002595
